# Binding between Saikosaponin C and Human Serum Albumin by Fluorescence Spectroscopy and Molecular Docking

**DOI:** 10.3390/molecules21020153

**Published:** 2016-01-28

**Authors:** Yi-Cun Chen, Hong-Mei Wang, Qing-Xia Niu, Dan-Yan Ye, Guo-Wu Liang

**Affiliations:** 1 Key Immunopharmacology Laboratory of Guangdong Province, Department of Pathophysiology, Institute of Inflammation and Immune Diseases, Shantou University Medical College, Guangdong 515041, China; chenyicun@yeah.net (Y.-C.C.); junebulu@163.com (H.-M.W.); gwliangizzy@163.com (G.-W.L.); 2 Department of Pharmacology, Traditional Chinese Medicine Laboratory, Shantou University Medical College, Guangdong 515041, China; 15917907394@163.com

**Keywords:** saikosaponin C, human serum albumin, static quenching, *Radix bupleuri*, molecular docking, Chaihu

## Abstract

Saikosaponin C (SSC) is one of the major active constituents of dried *Radix bupleuri* root (Chaihu in Chinese) that has been widely used in China to treat a variety of conditions, such as liver disease, for many centuries. The binding of SSC to human serum albumin (HSA) was explored by fluorescence, circular dichroism (CD), UV-vis spectrophotometry, and molecular docking to understand both the pharmacology and the basis of the clinical use of SSC/Chaihu. SSC produced a concentration-dependent quenching effect on the intrinsic fluorescence of HSA, accompanied by a blue shift in the fluorescence spectra. The Stern-Volmer equation showed that this quenching was dominated by static quenching. The binding constant of SSC with HSA was 3.72 × 10^3^ and 2.99 × 10^3^ L·mol^−1^ at 26 °C and 36 °C, respectively, with a single binding site on each SSC and HSA molecule. Site competitive experiments demonstrated that SSC bound to site I (subdomain IIA) and site II (subdomain IIIA) in HSA. Analysis of thermodynamic parameters indicated that hydrogen bonding and van der Waals forces were mostly responsible for SSC-HSA association. The energy transfer efficiency and binding distance between SSC and HSA was calculated to be 0.23 J and 2.61 nm at 26 °C, respectively. Synchronous fluorescence and CD measurements indicated that SSC affected HSA conformation in the SSC-HSA complex. Molecular docking supported the experimental findings in conformational changes, binding sites and binding forces, and revealed binding of SSC at the interface between subdomains IIA-IIB.

## 1. Introduction

Serum albumin is the most abundant plasma protein, being present in plasma at a concentration of approximately 40 mg·mL^−1^ (~0.6 mM) [1]. In addition to blood, about 60% of the total albumin is also found in tissues and secretions [2]. As a carrier protein, HSA plays an essential role in the binding of drugs, as well as endogenous and exogenous substances, such as fatty acids, bilirubin, and metal ions, in the bloodstream [3,4]. The organic small molecules and proteins interact with each other by means of basic amino acid residues on the protein, including arginine, lysine and histidine residues, and *N-*terminal amino groups [5]. The interaction between drugs and HSA directly affects the absorption, distribution, free drug concentration, and therapeutic effects [6]. Therefore, drug-HSA coupling has been widely investigated in the life sciences, chemistry, pharmacology and clinical medicine [7,8,9,10]. In practice, it is very difficult to directly measure drug-protein binding *in vivo*. In recent years, the binding properties of HSA with drugs have extensively been characterized *in vitro* by fluorescence spectroscopy.

Chaihu, derived from *Bupleurum chinense* DC or *Bupleurum scorzoneraefolium* Willd, has been widely administered for many centuries as a well-known medicinal herb in China, Japan and other Asian countries [11]. The contemporary clinical applications for Chaihu include a variety of diseases, especially liver diseases, for instance, jaundice, hepatitis and liver cirrhosis [12,13,14]. Saikosaponin C (SSC, CAS Registery Number: 20736-08-7, Figure 1), a triterpenoid saponin, is one of the major active ingredients in Chaihu. Recently, it has been recently reported to exert various biological effects. SSC efficiently inhibits hepatitis and apoptosis, and facilitates the growth, migration and angiogenesis of endothelial cells [12,15,16]. Although SSC is one of the major components in Chaihu, and possesses important biological functions, the interaction between SSC and HSA is still unknown.

In the present study, SSC purified from *Bupleurum chinense DC* was employed as a quencher for HSA fluorescence. Fluorescence, circular dichroism (CD) spectroscopy, site marker competitive experiments, UV absorption, theoretical analysis, and molecular docking studies were performed for the first time in an attempt to investigate the interaction and mechanisms of SSC with HSA. This study should provide useful information about the pharmacology and role of SSC/Chaihu in clinical medicine.

**Figure 1:**
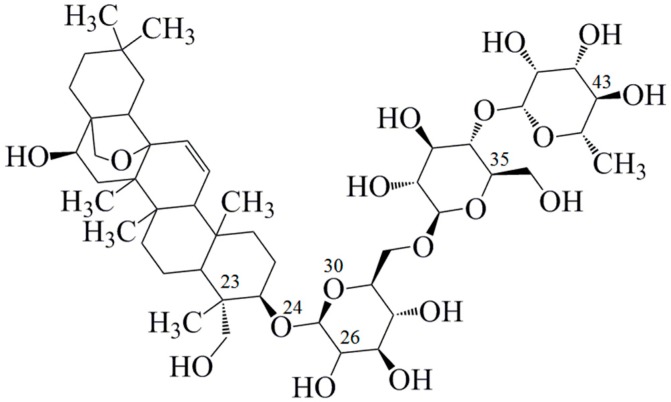
Chemical structure of saikosaponin C (C_48_H_72_O_17_, molecular weight 920, numbered using ChemDraw software).

## 2. Results and Discussion

### 2.1. Fluorescence Quenching of HSA by SSC

**Figure 2:**
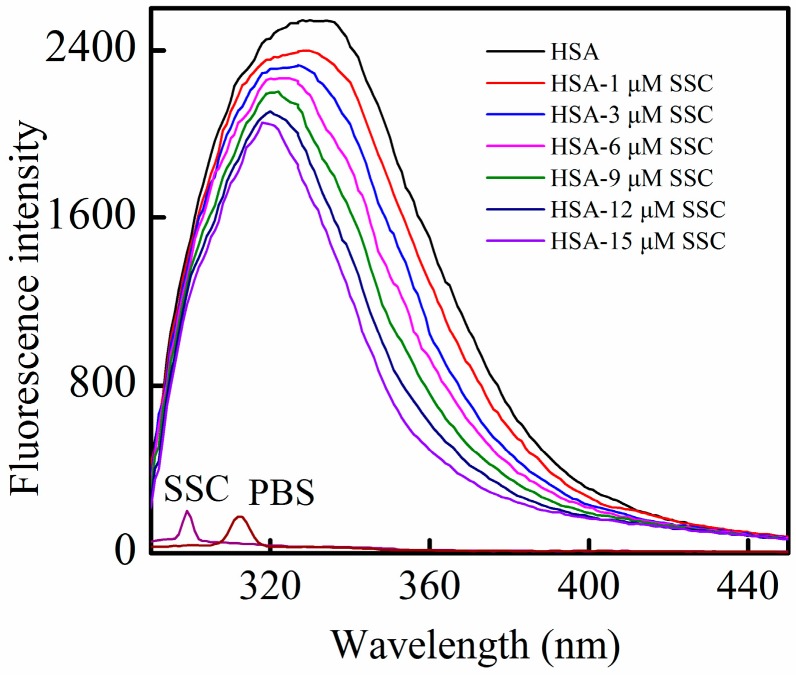
Fluorescence quenching of HSA in the presence of SSC. The observations were performed at 26 °C. λ_ex_ = 280 nm; C_HSA_ = 2 μM; SSC, saikosaponin C.

HSA contains an endogenous fluorophore. At a concentration of 2 μM, HSA showed a strong fluorescence at 26 °C, whereas SSC or the PBS control showed little (Figure 2). Little information is available on the interaction between saikosaponins and proteins. At an excitation of 280 nm, the maximum emission wavelength of HSA was 334 nm, which is in line with previous reports on the binding of other drugs to HSA [3]. Following SSC addition, the fluorescence intensity of HSA decreased regularly with the increase of SSC concentration. SSC did not affect the basic peak appearance of HSA. Importantly, SSC evoked a blue shift of the fluorescence peaks. At 15 μM SSC, the shift reached 16 nm**,** ranging from 334 to 318 nm, suggesting that SSC and HSA bind to each other to form an SSC-HSA complex. A blue shift of maximum emission wavelength reveals an increase of hydrophobic amino acid residues in the microenvironment of the fluorophores, and a red shift indicates an increase of polar residues [17,18]. Accordingly, the results as shown in Figure 2 imply that more hydrophobic amino acid groups are formed by the interaction between SSC and HSA.

### 2.2. SSC-Induced Static Quenching

Fluorescence quenching is classified into dynamic quenching and static quenching. Dynamic quenching is brought about by intermolecular collision between a quencher and fluorescent molecule at an excited state, in which effective collision and the quenching constant should increase with a rise in temperature. Dynamic quenching does not affect the structure and bioactivity of protein. Static quenching is derived from the intramolecular interaction of quenchers with fluorescent molecules at a ground state, which forms a new composite. In static quenching, the steadiness of new composites decreases with the rise in temperature, since high temperatures cause molecular diffusion and the dissociation of weakly bound complexes. Accordingly, dynamic quenching and static quenching can be distinguished by the response to temperature change. To examine the mechanism of SSC-induced quenching, the Stern-Volmer equation for dynamic quenching was first employed as follows [19,20]:

(1) $$\frac{F_{0}}{F}=1+K_{q}\tau_{0}\;\left[Q\right]=1+K_{sv}\left[Q\right]$$

where *F*_0_ and *F* stand for the fluorescence intensities in the absence and presence of the quencher, respectively. The average lifetime of fluorescent molecules without quenchers, τ_0_, is taken as 10^−8^ s [21]. *K_sv_*, known as the Stern-Volmer quenching constant, is the ratio of the bimolecular quenching rate constant to the decay rate constant of a single molecule (*K_sv_*= *K_q_*τ_0_). *[Q]* is the quencher concentration. *Kq* is the quenching rate constant of 2.0 × 10^10^ L·mol^−1^·S^−1^, which is the limiting diffusion rate constant for the quencher and biological molecule complex [7]. The constant at both 26 °C and 36 °C was much higher than the criterion of 2.0 × 10^10^ L·mol^−1^·S^−1^, indicating that static quenching is mainly responsible for SSC-induced fluorescence quenching of HSA (Table 1).

**Table 1:** Quenching reactive constants of HSA by SSC.

Fluorescence	T (°C)	Detection	*Kq* (L·mol^−1^·S^−1^)	R
Conventional	26	λ_ex_ = 280 nm	4.19 × 10^1^^2^	0.996
	36	λ_ex_ = 280 nm	1.17 × 10^1^^2^ *	0.965
Synchronous	26	∆λ = 15 nm	2.97 × 10^1^^1^	0.963
	26	∆λ = 60 nm	2.89 × 10^1^^2^	0.995

R, linear correlation coefficient; * *p* < 0.05 compared with conventional fluorescence at 26 °C by the paired *t-*test.

**Figure 3:**
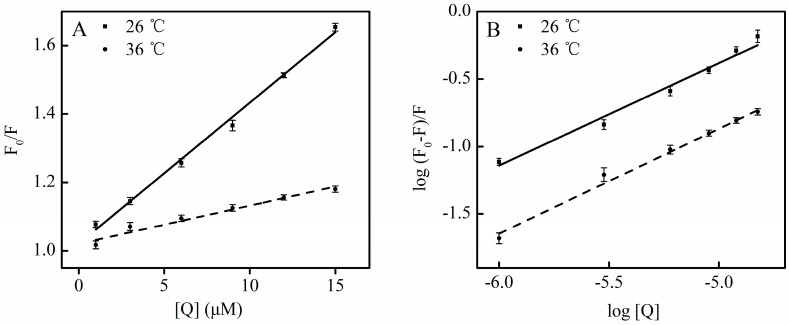
Plots for the interaction of SSC with HSA. Experimental conditions were as described in Figure 2. (**A**) Stern-Volmer plots for the binding of SSC to HSA; (**B**) Double-reciprocal plots for log (F_0_ − F)/F *vs.* log [Q]; F_0_, HSA fluorescence intensities without the quencher; F, HSA fluorescence intensities with the quencher. [Q], SSC at 0, 1, 3, 6, 9, 12 and 15 μM, respectively.

Moreover, as calculated from Equation (1), the *Kq* for SSC at 26 °C was much above the *Kq* at 36 °C, confirming that static quenching contributes to SSC-induced quenching of HSA. A plot (Figure 3), according to the findings in Figure 2, the Stern-Volmer equation and Table 1 shows good linearity and correlation coefficients, which supports static quenching created by SSC.

### 2.3. SSC-Induced Conformational Change in HSA

#### 2.3.1. Investigation of Synchronous Fluorescence

**Figure 4:**
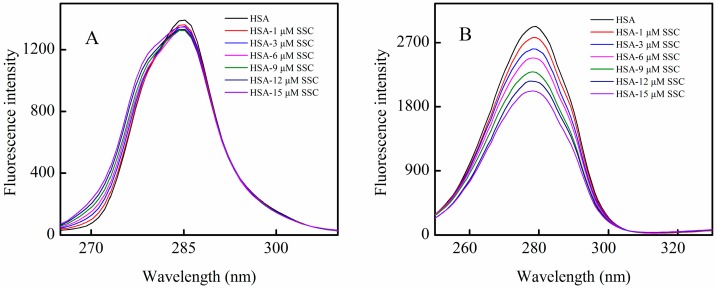
Synchronous fluorescence spectra of the SSC-HSA system. The experimental conditions were as described in Figure 2. A, Δλ = 15 nm for tyrosine; B, Δλ = 60 nm for tryptophan. C_HSA_ = 2 μM.

HSA fluorescence mainly comes from tyrosine or tryptophan residues. Δλ = 15 nm or Δλ = 60 nm is characteristic of fluorescence spectra by tyrosine or tryptophan residues, respectively. To clarify the mechanism of SSC-induced quenching of HSA, synchronous fluorescence spectrophotometry was used to obtain fluorescence spectra for tyrosine or tryptophan residues in HSA. At Δλ = 15 nm, the fluorescence intensity decreased slightly, and a blue shift of about 2 nm was observed at maximum emission wavelength when SSC concentrations increased (Figure 4A). A blue shift of the ascending branch of fluorescence spectra could be observed, and a blue shift of 4 nm, from 272 to 268 nm, occurred at a maximum point of shift (Figure 4A). These results revealed that SSC induces a conformational change in HSA. As shown in Figure 4B, the peak fluorescence at Δλ = 60 nm declined with SSC concentration. Also, a blue minor shift of 1 nm (from 279 to 278 nm) was produced at the maximum emission. These observations revealed that both tyrosine and tryptophan are likely involved in the binding reaction between SSC and HSA. The microenvironmental polarity around amino acid residues can be judged by the shift in the fluorescence spectra of amino acid residues closely related to the protein conformational changes [18]. The blue shift demonstrates that the related residues within HSA are closer, and the range of extension of the peptide chain becomes reduced. Subsequently, the microenvironmental polarity surrounding the residue is converted from hydrophilicity to hydrophobicity, folding the peptide chain and destroying its ordered structure in order to form a new conformation [22]. The changes of spectra for tyrosine or tryptophan were consistent with fluorescence spectra for HSA (Figure 2). Similar to HSA, the Kq at both ∆λ = 15 nm and ∆λ = 60 nm was also over the criterion of 2.0 × 10^10^ L·mol^−1^·S^−1^, suggesting that both tyrosine and tryptophan residues are most likely associated with static quenching. Additionally, a strong correlation for ∆λ = 15 nm or ∆λ = 60 nm was observed in Table 1, which supports SSC-mediated static quenching.

The minor blue shift is likely due to the fact that no direct interaction occurs between SSC and fluorophore in HSA. That is, tyrosine or tryptophan residues are indirectly micro-perturbed when SSC directly binds to the other amino acid residues surrounding SSC, leading to HSA conformation alterations. In order to investigate if there is a direct interaction between SSC and tyrosine or tryptophan residues, the corresponding synchronous fluorescence measurements were performed as described in Section 3.3.3. The results showed that there was no difference, on synchronous fluorescence spectra, between tyrosine or tryptophan alone and an SSC complex with each residue (data not shown), suggesting that the direct binding of SSC with the two residues did not exist.

#### 2.3.2. HSA Conformational Change by CD Measurements 

**Figure 5:**
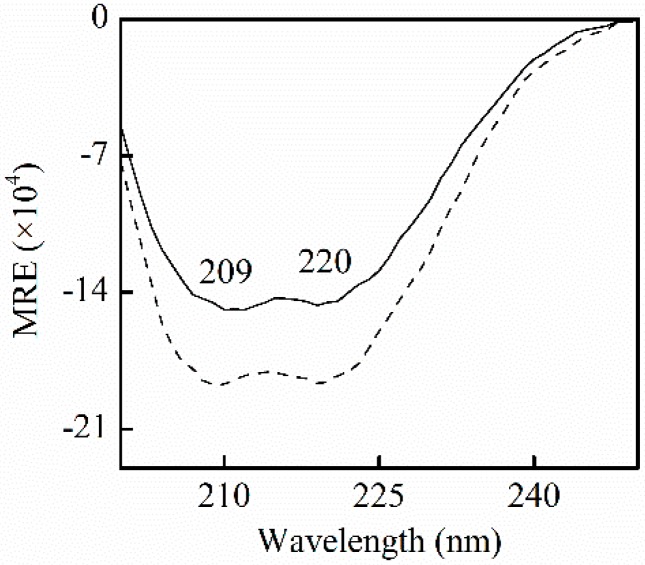
CD spectra for HSA in the absence and presence of SSC in pH7.4, 0.5 M Tris-Cl buffer. C_HSA_ = 0.42 μM; C_SSC_ = 0.42 μM. A solid line stands for free HSA. A dotted line is for HSA-SSC. MRE, mean residue ellipticity.

CD was applied to clarify the conformational effect of SSC on HSA. The α-helical content in free HSA and the HSA-SSC complex can be calculated by the following equations [23]:

(2) $$MRE=\frac{CD\left(m\;deg\right)}{10C_{p}nl}$$

(3) $$\alpha -helix\left(\%\right)=\frac{-{\left(MRE\right)}_{209}-4000CD\left(m\;deg\right)}{33000-4000}\times 100$$

where mean residue ellipticity (MRE)_209_, *C_p_*, *n*, 33,000, and *l* is the mean residue ellipticity observed at 209 nm, HSA concentrations, the number of amino acid residues, the MRE of a pure α-helix at 209 nm, and the path length, respectively. The 4000 is the MRE of the β-form and random coil conformation cross at 209 nm. Figure 5 shows the negative absorption at near 209 nm and 220 nm, the α-helical characters of proteins, which contributes to π-π* and n-π* electron transfer for the peptide bond of α-helix, respectively. The negative peak at 209 nm slightly shifted to the left. No shift of the peak was observed at 209 nm. The content of α-helix and β-turn increased by 4.9% (from 55.9% to 60.8%), and 4.8% (from 5.7% to 10.5%), and β-sheet and random coil decreased 2.3% (from 19.1% to 16.8%), and 3.2% (from 16.0% to 12.8%), respectively. In accordance with the blue shift by fluorescence measurements, the changes indicate that the extension of the peptide chain is partly reduced following the binding of SSC to HSA. This is perhaps due to the fact that SSC perturbs π-π* and n-π* responses, and provokes the rearrangement of the polypeptide network.

Few observations on the binding of SSC to HSA are available. α-helices and β-sheets in Figure 5 are consistent with CD spectroscopic studies for free HSA [24]. β-turns and random coils differed little from some reports [24,25], and could be accounted for by minor differences in experimental conditions. As described by our synchronous fluorescence study, the results indicate that SSC can interact with HSA, and influence its secondary structure.

### 2.4. Binding Constants, Sites and Forces between SSC and HSA

#### 2.4.1. Binding Constants and Numbers of Binding Sites

The *K_a_* for high affinity binding is higher than 10^4^ L·mol^−1^ in order of magnitude. The *K_a_* for low affinity binding is lower than 10^3^ L·mol^−1^ in order of magnitude [24]. The *K_a_* is obtained based on Equation (4) [19]:

(4) $$log\frac{\left(F_{0}-F\right)}{F}=logK_{a}+nlog\left[Q\right]$$

The resulting *K_a_* value was 3.72 × 10^3^ and 2.99 × 10^3^ L·mol^−1^. The number of binding sites was 0.79 and 0.83 at 26 °C and at 36 °C, respectively (Table 2). These indicate that SSC binds to HSA with low affinity, and the binding is weak and easily reversed. No statistical difference in the *K_a_* value was observed between 26 °C and 36 °C, suggesting that the quenching efficiency of SSC to HSA was constant at the tested temperatures. These reveal that SSC can be bound, transported, and released by HSA into target tissues.

**Table 2:** Binding reactive parameters of HSA by SSC.

Fluorescence	T (°C)	Detection	*K_a_* (L·mol^−1^)	R	*n*
Conventional	26	λ_ex_ = 280 nm	3.72 × 10^3^	0.983	0.79
	36	λ_ex_ = 280 nm	2.99 × 10^3^	0.959	0.83
Synchronous	26	∆λ = 15 nm	0.90 × 10^2^	0.986	0.57
	26	∆λ = 60 nm	1.85 × 10^3^	0.989	0.77

*K_a_*, binding constant; R, linear correlation coefficient; *n*, the number of binding sites.

#### 2.4.2. Identification of Warfarin and Ibuprofen Binding Sites

Crystallographic analyses show that HSA, a 585 amino acid residue monomer protein, contains three homologous α-helical domains (domains I–III), each of which includes subdomain A and B, to comprise six subdomains, IA, IB, IIA, IIB, IIIA, and IIIB. The two subdomains in each domain constitute a hydrophobic cavity [25]. HSA has a limited number of binding sites for a variety of ligands that are typically bound reversibly [1]. Crystal structure analyses also indicate that the two principal regions of ligand binding sites in albumin, site I and site II, are positioned in hydrophobic cavities. Site I, located within subdomain IIA, preferentially integrates with bulky heterocyclic ligands, such as warfarin and, bilirubin [9,26]. Site II, located within subdomain IIIA, preferentially recognizes aromatic compounds, and is more active in the accommodation of ligands, for example, digitoxin, ibuprofen, and tryptophan [27].

**Figure 6:**
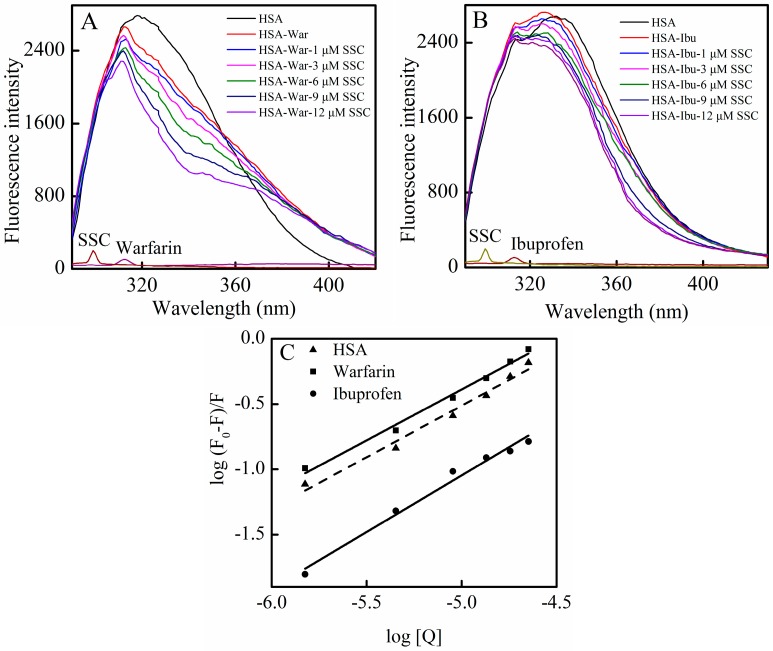
Effect of site marker probes on the SSC-HSA system. The experiment was carried out at C_HSA_ = 2 μM, T = 26 °C, pH = 7.4, and λ_ex_ = 280 nm. (**A**) C_warfarin_ = 0.025 M; (**B**) C_ibuprofen_ = 0.25 M; (**C**) Double-reciprocal plots for log (F_0_ − F)/F *vs.* log [Q]; [Q], C_SSC_ = 0, 1, 3, 6, 9, and 12 μM. War, Warfarin; Ibu, Ibuprofen.

In order to identify SSC binding sites on the region of HSA molecule, site competitive binding experiments have been commonly carried out. Warfarin (an anticoagulant drug, a marker of site I) and ibuprofen (a nonsteroidal anti-inflammatory agent, a marker of site II) were utilized in the present experiment. Following the addition of SSC into the HSA-warfarin solution, the fluorescence intensity of HSA became significantly reduced (Figure 6A). A shift in fluorescence spectra at the maximum emission wavelength was not found in the warfarin mixture system, while the minor blue shift was observed at ∆λ = 15 nm or ∆λ = 60 nm in Figure 4, suggesting that the binding of warfarin to HSA inhibits the formation of hydrophobic structures surrounding tyrosine or tryptophan residues in HSA-SSC complex. As shown in Figure 6B, following the addition of SSC into the HSA-ibuprofen solution, HSA fluorescence intensity weakened dose-dependently. In addition, ibuprofen induced a blue shit of 11 nm (from 336 nm to 325 nm) in the ibuprofen mixture system, indicating that the hydrophobicity of amino acid residues arises around ibuprofen. The competitive effect of ibuprofen on HSA binding reveals that SSC mainly binds site II (subdomain IIIA) in HSA.

According to the Equation (4), the corresponding plot can be obtained from the fluorescence data (Figure 6C) [28]. The *K_a_* in the warfarin or ibuprofen mixture was 4.38 × 10^4^ L·mol^−1^ or 2.22 × 10^3^ L·mol^−1^, respectively (Table 3). Although the binding constants in the study system are low, lower binding constants (about 10^3^ L·mol^−1^) have also been reported for protein–ligand interactions [29]. Commonly, weak binding to the protein results in a shorter lifetime or poor distribution of drugs in plasma, whereas the strong binding reduces the levels of free plasma drugs [10]. The results showed that the *K_a_* of the warfarin complex was much higher than that of SSC-HSA binding, whereas the *K_a_* of the ibuprofen complex is below the binding constant of SSC and HSA (Table 3). Confusedly, the *K_a_* in the mixture system is higher in the presence of warfarin. Obviously, the effect requires further elucidation in the future. Albumin-binding events play an essential role in the transportation, distribution and free fraction of drugs. Free drugs are responsible for pharmacological activities [30]. The calculated results show that the effect of warfarin or ibuprofen is of practical significance in clinical application. If warfarin is given at the same time with SSC, the warfarin-HSA binding will be able to regulate the binding of SSC and HSA, extend the retention time in plasma, decrease free concentration, and inhibit the effect of SSC/Chaihu. If ibuprofen combines with SSC at the same time, the ibuprofen-HSA interaction can influence SSC and HSA binding, elevate the free concentration, and enhance the effect of SSC/Chaihu. Warfarin, a coumarin representative, and other coumarin anticoagulants, such as acenocoumarol and phenprocoumon, widely exist, not only among Chinese herbal medicines, but also among prescribed drugs worldwide [31]. Ibuprofen, a classical anti-inflammatory drug, and its derivatives are also among the most common prescribed drugs in the world [32]. Chaihu enriching SSC is often prescribed as a major herbal medicine both in some classical Chinese medicinal formulae, such as “xiao yao powder”, and in any prescription, such as Chaihu and *Scutellaria Baicalensis*, Chaihu and peony, and Chaihu and *Liquorice* [33]. Inevitably, with the extensive application of Chaihu in clinical practice, SSC commonly interacts not only with co-administered medicinal herbs, but also likely with co-treated modern drugs. In short, the effect of SSC/Chaihu is likely weakened by warfarin-class drugs, whereas the effect of SSC/Chaihu is likely strengthened when ibuprofen-class drugs and SSC/Chaihu are co-administered.

**Table 3:** Binding reactive parameters in site competitive experiments.

System	Equation	*K_a_* (L·mol^−1^) ± SD	R
Warfarin	Y = 0.78X + 3.64	4.38 × 10^4^ ± 11.65 *	0.991
Ibuprofen	Y = 0.87X + 3.35	2.22 × 10^3^ ± 3.75 *	0.985
HSA-SSC	Y = 0.79X + 3.57	3.72 × 10^3^ ± 4.76	0.983

*K_a_*, binding constant; R, linear correlation coefficient; SD, standard deviation; * *p* < 0.05 compared with HSA-SSC control by the paired *t-*test.

#### 2.4.3. Binding Forces between SSC and HSA

The binding forces between a small molecule and a macromolecule can include several types of interactions, such as hydrogen bonds, van der Waals forces, electrostatic forces, and hydrophobic interactions [34,35]. The thermodynamic parameters, enthalpy change (∆*H*) and entropy change (∆*S*), are the major indicators to confirm binding modes. Based on a number of experimental results, Ross summarized the thermodynamic relationship between proteins and small molecules as follows: ∆*H* < 0 and ∆*S* < 0 indicates van der Waals force or hydrogen bond formation; ∆*H* < 0 and ∆*S* > 0 implies an electrostatic force; and ∆*H* > 0 and ∆*S* > 0 reflects hydrophobic interaction [36]. 

The enthalpy change can be considered as a constant when the effect of tested temperatures on binding constants is not statistically significant (Table 2). From the temperature dependence of the binding constants, the parameters involved in the process were calculated using the following equations (Table 4) [37,38,39]:

(5) $$ln\frac{K_{a2}}{K_{a1}}=\frac{∆H}{R}\left(\frac{1}{T_{1}}-\frac{1}{T_{2}}\right)$$

(6) $$∆G=-RTlnK_{a}$$

(7) ∆G=∆H−T∆S

where *K_a_* is the binding constant calculated by Equation (4), and *R* is the gas constant. As shown in Table 4, the negative value of the free energy change (∆*G*) means a spontaneous binding process. Both negative enthalpy and entropy values reveal that van der Waals interactions and hydrogen bonds play a major role in the binding process between SSC and HSA (Table 4). Therefore, the interaction between SSC and HSA is a forward reaction involving the reduction of free energy change, and enthalpy change and entropy change at a low temperature, which is mainly driven by enthalpy because the entropy value was negative and unfavorable for the binding process.

**Table 4:** Thermodynamic parameters of SSC-HSA interaction.

Fluorescence	T (°C)	λ_ex_	∆*G* (kJ·mol^−1^)	∆*H* (kJ·mol^−1^)	∆*S* (J·mol^−1^·K^−1^)
Conventional	26	280 nm	−20.34	−47.34	−90.31
	36	280 nm	−19.52		−90.04

### 2.5. Energy Transfer between HSA and SSC

Energy transfer, one of the most conspicuous intermolecular events of the interaction between drugs and albumin, is an electrodynamic process in which a primary excited-state donor can transfer energy to its neighbors through a non-radiative dipole-dipole coupling [38]. The process takes place under the following conditions. First, the donor can produce fluorescent light. Second, there is a fair amount of overlap between the fluorescence emission spectra of the donor and the UV-vis absorbance spectra of the acceptor. Additionally, the distance between the donor and the acceptor approaches less than 8 nm if fluorescence emitted from a donor can be absorbed by an acceptor [40,41]. The phenomenon of energy transfer is associated not only with the distance between the donor and the acceptor, but also with the critical energy transfer distance. According to the Förster mechanism of non-radiative energy transfer, the energy transfer efficiency, and the distance between the donor (HSA) and the acceptor (SSC) can be calculated by the following equation [38]:

(8) $$E=1-\frac{F}{F_{0}}=\frac{R_{0}^{6}}{R_{0}^{6}+r^{6}}$$

where *E* represents the efficiency of transfer between the donor and the acceptor, which can be measured experimentally by the fluorescence emission from donors with (*F*) and without the acceptor (*F*_0_), and normalized to the same donor concentration [17]. Here, *r* is the average distance between donor and acceptor. *R*_0_ is the critical distance when the efficiency of transfer is 50%, which relates to the quantum yield of the donor, the quenching coefficient of the acceptor, the overlap between the fluorescence emission from the donor and absorption spectra from the acceptor, and the orientation of the fluorophore. The calculation for *R_0_* is described in the following expression [42]:

(9) $$R_{0}^{6}=8.79\times 10^{-25}K^{2}n^{-4}J\varphi$$

where *K*^2^, the spatial orientation factor between the emission dipole of the donor and the absorption dipole of the acceptor, is the least certain parameter in the calculation of the critical transfer distance. If both the donor and the acceptor rotate rapidly without any orientation in a supposition, then *K*^2^ = 2/3. If only the donor is free to spin, then *K*^2^ ranges from 1/3 to 4/3 [43]. Here, *n* is the average refractive index of medium in the wavelength range where spectral overlap is significant, and φ is the fluorescence quantum yield of the donor. In the present work, *K*^2^ = 2/3, *n* = 1.336, φ = 0.118 [44]. *J* is the overlap integral of the fluorescence emission spectrum of the donor and the absorption spectrum of the acceptor, given by the equation below [22]:

(10) $$J=\frac{\sum^{\text{​}}F(\lambda )\varepsilon \left(\lambda \right)\lambda^{4}∆\lambda }{\sum^{\text{​}}F(\lambda )∆\lambda }$$

**Figure 7:**
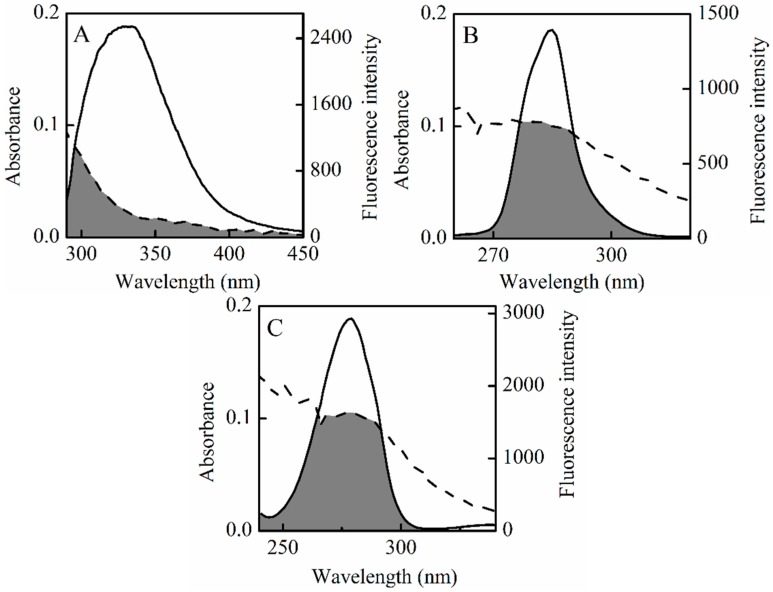
Overlap of the UV absorption spectra of SSC and the fluorescence emission spectra. Dotted line is the UV absorption spectra of 3 mM SSC. (**A**–**C**) is the fluorescence emission spectra of C_HSA_ = 2 μM, ∆λ = 15 nm or ∆λ = 60 nm, respectively.

In Equation (10), *F*(λ) is the fluorescence intensity of the fluorescent donor at wavelength *λ*, and is dimensionless. Here, ε(λ) is the molar absorption coefficient of the acceptor at wavelength *λ.* The overlap integral (*J*) could be evaluated by integrating the spectra in Figure 7. Based on these data, *J*, *R*_0_, *E* and *r* could be obtained between the donor and the acceptor (Table 5). The average distances from a donor to an acceptor in HSA, ∆λ = 15 nm, and ∆λ = 60 nm are all on the 2 to 8 nm scale, and 0.5*R_0_* < *r* < 1.5*R*_0_ [45], which indicates that non-radiative energy transfer occurs with high probability between HSA and SSC, and re-confirms that a static quenching interaction arises between SSC and HSA [36,46].

**Table 5:** Energy transfer parameters between SSC and HSA.

Fluorescence	T (°C)	Detection	*J* (cm^3^·L·mol^−1^)	*R*_0_ (nm)	*E* (J)	*r* (nm)
Conventional	26	λ_ex_ = 280 nm	6.54 × 10^−16^	2.14	0.23	2.61
	36	λ_ex_ = 280 nm	3.06 × 10^−16^	1.89	0.096	2.74
Synchronous	26	∆λ = 15 nm	1.14 × 10^−15^	2.34	0.037	4.03
	26	∆λ = 60 nm	2.65 × 10^−15^	2.69	0.19	3.45

### 2.6. Docking Results

**Figure 8:**
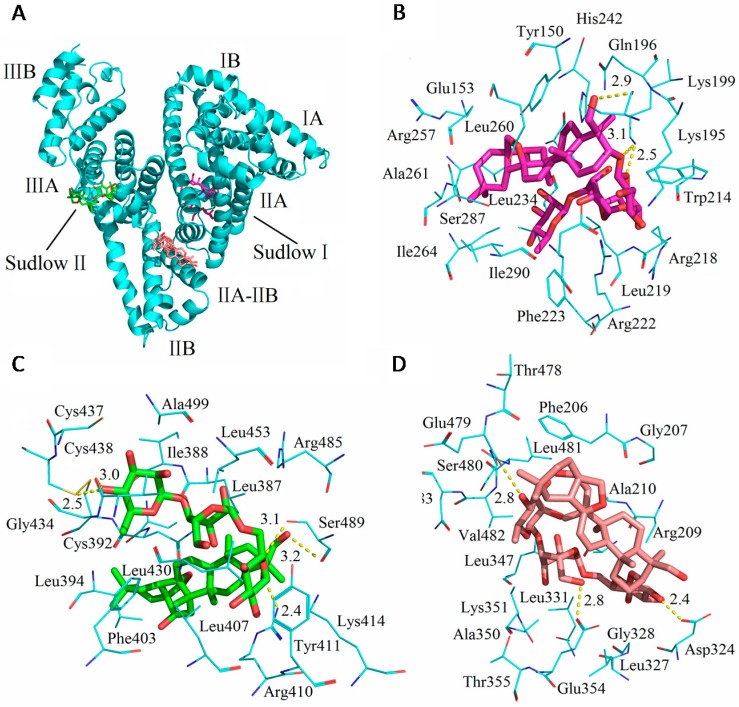
Optimal binding conformation of the SSC-HSA complex (**A**). Amino acid residues of Sudlow I (**B**); or Sudlow II (**C**) or IIA-IIB binding site (**D**) surrounding SSC within 4 Å. The cyan structure stands for HSA. The pink, green, or brown is for SSC, and a dotted line for hydrogen bonding.

Figure 8 shows the theoretical binding mode of SSC to HSA. Trp214, Tyr150 and Tyr411 were located surrounding the hydrophobic pockets in Figure 8B,C, and tyrosine or tryptophan residues were not found at the interface between sub-domain IIA-IIB in Figure 8D, which corresponds to fluorescence spectra in the study. As shown in Figure 8B, SSC adopted a hairpin conformation to binding inside the pocket of the Sudlow I binding site in subdomain IIA (site I). Only three hydrogen bonds were observed between SSC and Sudlow I. Figure 8C demonstrates that SSC docked into the subdomain IIIA (site II, Sudlow II), and five hydrogen bonds occurred between SSC and Sudlow II. Based on the information provided by the PBD databank, SSC was also docked to the IIA-IIB binding site of HSA (Figure 8D). The glucoside group of SSC fit at the bottom of the HSA pocket and a high density of van der Waals contacts, whereas the bridged-ring group of SSC was positioned within the interface between sub-domain IIA-IIB, and made only a few contacts. Moreover, only three hydrogen bond interactions were observed between SSC and HSA.

The above analysis fits extremely well with the site experimental results and binding forces. Detailed analysis was shown in Table 6. To a greater degree, hydrogen bonds increase the stability of SSC-HSA complexes. Accordingly, it is inferred that the SSC-Sudlow II complex is likely the most stable among the three binding modes. Consistent with the site experimental findings, the present docking studies indicate that SSC binds to site I and II. Moreover, the study found binding of SSC to the interface between subdomain IIA-IIB. These results indicate that docking analysis is helpful for providing a structural basis for the interaction between HSA and SSC.

**Table 6:** Atoms and amino acid residues involved in the hydrogen bonds between SSC and HSA.

Subdomain	Amino Acid Residues/HSA	Atoms/SSC *	Length
Sudlow I	Ser192	CH_2_OH(19)	2.9 Å
	Lys199	O(23)	3.1 Å
	Lys199	O(25)	2.5 Å
Sudlow II	Arg-410	O(23)	2.4 Å
	Ser489	CH_2_OH(19)	3.1 Å
	Ser489	O(25)	3.2 Å
	Cys438	OH(45)	2.5 Å
	Val433	OH(45)	3.0 Å
IIA-IIB	Asp324	OH(26)	2.4 Å
	Glu354	CH_2_OH(37)	2.8 Å
	Glu479	OH(45)	2.8 Å

*, see Figure 1.

## 3. Materials and Methods

### 3.1. Materials

HSA (approx. 99.0% purity by electrophoresis), tyrosine and tryptophan (both 98.0% purity by TLC) were purchased from Sigma (St Louis, MO, USA). SSC (98.0% purity by HPLC) was obtained from Biopurify Phytochemical Ltd. (Chengdu, Sichuan, China). Ibuprofen and warfarin (both 99.7% purity) were supplied by the National Institute for Control of Pharmaceutical and Bioproducts (Beijing, China). All other reagents were of analytical grade, and used without further purification. Sample masses were accurately weighed on a microbalance (Sartorius, Gottingen, Germany) with a resolution of 0.01 mg. All pH measurements were made with a pH meter (Sartorius, Gottingen, Germany).

### 3.2. Absorbance Measurements

The UV-vis absorption spectrum was recorded at room temperature on a NanoDrop 2000c spectrophotometer (Thermo, Waltham, MA, USA) equipped with 1.0 cm quartz cells. Spectrum data points were collected from 280 to 450 nm. 

### 3.3. Fluorescence Measurements

#### 3.3.1. Conventional Fluorescence Measurements for SSC-HSA Binding

All fluorescence measurements were recorded on a F-7000 spectrofluorimeter (Hitachi, Tokyo, Japan) equipped with 1.0 cm quartz cells. The widths of the fluorescence excitation slit and emission slit were fixed at 2.5 nm for all experiments. Scan speed was set at 1200 nm·min^−1^. The excitation wavelength was set at 280 nm [47]. In each assay, 3.0 mL HSA at a concentration of 2 μM was added into the quartz cell. Subsequently, the HSA solution was titrated by successive additions of a 3 mM stock solution of SSC, using a microsyringe, to a final SSC concentration of 0, 1, 3, 6, 9, 12 and 15 μM. Fluorescence intensities were measured at 26 °C (=299 K) and 36 °C (=309 K). All samples were prepared in 0.01 M phosphate buffer saline (PBS) at pH 7.4. Each experiment was repeated three times. Origin 8 SR2 Software (OriginLab, Nothampton, MA, USA) was used for plotting and calculation.

#### 3.3.2. Synchronous Fluorescence Measurements for SSC-HSA Binding

Synchronous fluorescence spectroscopy involves the simultaneous scanning of excitation and emission monochromators at a fixed wavelength difference (Δλ = λ_em_ − λ_ex_), with good selectivity and sensitivity, and less interference. The excitation and emission slit widths were fixed at 2.5 nm. The scan speed was set at 240 nm·min^−1^. In each assay, 3.0 mL HSA at 2 μM was titrated by successive additions of 3 mM SSC (concentration from 0 to 15 μM). Synchronous fluorescence was always performed at 26 °C. The spectrum data points were collected with Δλ = 15 nm for tyrosine or Δλ = 60 nm for tryptophan. Each experiment was repeated three times.

#### 3.3.3. Synchronous Fluorescence Measurements for SSC-Tyrosine or SSC-Tryptophan Binding

For tyrosine or tryptophan, 3.0 mL solutions, at 10 μM concentration, were titrated by successive additions of 3 mM SSC solution. The other procedures were the same as Section 3.3.2.

#### 3.3.4. Site Marker Competitive Fluorescence Experiments

In the presence of two site markers, warfarin and ibuprofen, binding site studies between SSC and HSA were carried out by conventional fluorescence, as described in Section 3.3.1. Before displacement, 2.0 mL of 2 μM HSA was incubated, at 26 °C for 30 min, with 0.25 M ibuprofen or 0.025 M warfarin in PBS at pH 7.4. Then, SSC solution was titrated into a 1.0 cm quartz cuvette by successive additions. For warfarin or ibuprofen solution alone, the fluorescence data were collected in similar fashion as the mixture system. Each experiment was repeated three times.

#### 3.3.5 Correction of the Internal Filter

To avoid the effect of the internal filter in the SSC-HSA system on the fluorescence data, the following formula was utilized [8]:

(11) $$F_{cor}=F_{obs}\times 10^{\left(A_{ex}+A_{em}\right)/2}$$

where *F_cor_* and *F_obs_* are the fluorescence intensities after and before the correction. *A_e__x_* and *A_em_* are the absorbance at the excitation and emission, respectively.

### 3.4. CD Spectroscopy 

CD measurements of HSA with and without SSC were recorded using a MOS 450 automatic spectropolarimeter (BioLogic Science Instruments, Claix, France) equipped with 1.0 cm quartz cells at 26 °C, over the scan range of 200–350 nm, at a scan rate of 30 nm/min with a response time of 4 s. Data were corrected using the signal of the buffer solution.

### 3.5. Molecular Docking

Molecular docking was performed to investigate the binding mode of SSC to HSA by means of Autodock Vina software (version 1.1.2). The three-dimensional (3D) coordinate of the HSA (PDB ID: 2XVU) was downloaded from the Protein Data Bank (http://www.rcsb.org/pdb) [48]. The Auto Dock Tools version 1.5.6 software (http://mgltools.scripps.edu) was employed to generate the docking input files. Ligand structure was prepared for docking by merging non-polar hydrogen atoms and defining rotatable bonds. In order to increase the docking accuracy, the value of exhaustiveness was set to 20. For Vina docking, the default parameters were used as described in the Autodock Vina manual unless otherwise specified. The top ranked pose as judged by the Vina docking score was subject to visually analysis by using PyMOL 1.7 software (http://www.pymol.org/). 

## 4. Conclusions

SSC can specifically bind to HSA and quench HSA’s intrinsic fluorescence by a static mechanism. The Stern-Volmer constant is inversely correlated with temperature, indicating that the formation of SSC-HSA complexes is predominantly responsible for the quenching. Thermodynamic parameters indicate that the binding process occurs spontaneously, and that hydrogen bonds and Van der Waals forces play a major role. The calculated binding constants and binding mechanism is consistent with the experimental data. Based on the Förster theory, non-radiation energy transfer can occur. Experimental site studies suggest that SSC can bind to site I (subdomain IIA) and site II (subdomain IIIA) of HSA. The results obtained from synchronous fluorescence, and CD spectroscopy demonstrate that the HSA conformation is affected by SSC when the two bind. Molecular docking studies further confirm the experimental findings in conformational change, binding site and binding force induced by SSC. Besides, SSC is also docked to the interface between subdomain IIA and IIB. Notably, SSC action may be obviously weakened by the co-administration of SSC/Chaihu and warfarin-class drugs, whereas SSC action may be promoted when ibuprofen-class drugs and SSC/Chaihu are co-administered at the same time. 
